# Psychosis in a Complex Medical Landscape: Diagnostic Challenges Posed by Systemic Lupus Erythematosus, Antiphospholipid Syndrome, Breast Cancer, and Temporal Lobe Resection

**DOI:** 10.7759/cureus.87266

**Published:** 2025-07-04

**Authors:** Khaled Alharmoodi, Jeremy Chan, Samuel Towell, Mateen Durrani

**Affiliations:** 1 Department of Psychiatry, Essex Partnership University NHS Foundation Trust, Colchester, GBR; 2 Department of Psychiatry, Colchester General Hospital, Colchester, GBR

**Keywords:** brain cancer, case report, chemotherapy, liaison psychiatry, neuropsychiatric systemic lupus erythematosus, psychosis

## Abstract

This study aimed to highlight the complex diagnostic process in a patient presenting with a new onset of both psychiatric and neurological signs and symptoms in the context of a complex medical background. A 60-year-old woman presented with psychosis, paranoia, disorientation, and choreiform movements, with a medical history of systemic lupus erythematosus (SLE), antiphospholipid syndrome (APS), active breast cancer, and a recent temporal lobe resection. The differential diagnoses considered included various neuropsychiatric conditions, such as neuropsychiatric SLE, steroid-induced psychosis, chemotherapy-induced neurotoxicity, and paraneoplastic syndrome, as well as primary psychotic disorder. The case illustrates the often inconclusive nature of various investigations, including autoimmune blood panels, magnetic resonance imaging, and electroencephalography, all of which failed to yield a definitive diagnosis. It also underscores the need for a multidisciplinary approach involving psychiatric and medical specialists, alongside regular observation and input from other ward staff, to optimally manage patients with such complex presentations.

## Introduction

An awareness of neuropsychiatric presentations of certain medical conditions is vital in psychiatric practice, particularly in liaison psychiatry, where one is often called to assist in the differentiation between medical and primary psychiatric presentations. Neuropsychiatric presentations are relatively common, with one study reporting that up to 40% of patients with systemic lupus erythematosus (SLE) experience a neuropsychiatric episode within a two-year period [1]. The most common time for patients to exhibit neuropsychiatric presentations was early in the disease process, at the onset of the study, highlighting the importance of considering neuropsychiatric presentations in any clinical encounter in psychiatry. It is further well documented that there is a significant iatrogenic psychiatric burden from patients receiving certain medications, particularly steroids, which have seen rates of psychiatric side effects as high as 57%, with severe events in as many as 6%, with a positive correlation seen between dose received and both frequency and severity of psychiatric side effects [2]. Additionally, chemotherapeutic agents are known to have their own psychiatric side effect profile, including cognitive disturbances, delirium, and psychosis [3,4]. Given the prevalence of neuropsychiatric presentations in certain medical conditions and their associated morbidities, psychiatrists and medical professionals would benefit from a heightened awareness of such presentations and their etiologies.

## Case presentation

The patient was a 60-year-old woman with a complex medical history, including breast cancer first diagnosed in 2009, managed with chemotherapy and radiotherapy. She experienced a recurrence in 2020 and continued to be under active treatment with chemotherapy. In December 2023, she developed temporal lobe metastases, which were surgically excised in January 2024, followed by adjuvant gamma knife radiosurgery. She was undergoing regular surveillance with triennial MRI scans. Additionally, she had a longstanding diagnosis of antiphospholipid syndrome (Hughes’ syndrome, 2001) and systemic lupus erythematosus (SLE, 1998), for which she was receiving hydroxychloroquine 200 mg once daily and prednisolone 10 mg once daily.

She presented to the emergency department in early 2025 following a three to four-month decline in mental state, initially characterized by insomnia and escalating anxiety. Her general practitioner diagnosed an anxiety disorder and prescribed promethazine, but within days, she developed acute psychiatric decompensation, manifesting as disorganized thought processes, paranoid ideation, and markedly bizarre behavior.

Obtaining a coherent history was profoundly challenging due to her acute psychotic state. She exhibited perseverative self-accusation, repetitively stating, “the same thing will happen” if discharged home. She articulated delusions of external control, insisting that her admission had been orchestrated by external forces and that she had “given up.” A preoccupation with inadequate mask usage emerged, which she attributed to her photosensitivity, alongside fragmented references to her husband’s illness and her perceived loss of cognitive autonomy. Her speech was disjointed and tangential, punctuated by self-reproach regarding her circumstances. She displayed marked paranoia and confusion, intermittently misidentifying clinicians as intruders in her home. During the assessment, she refused to engage, declaring, “You will contaminate me,” and recurrently asserted that her predicament was self-inflicted.

On examination, the patient presented as a frail, cachectic woman wearing three tightly layered surgical masks and noise-cancelling headphones, which she attributed to hyperacusis. Her behavior was markedly abnormal, characterized by bizarre, purposeless motor activity alternating between agitated pacing and episodes of frozen rigidity, accompanied by persistent choreoathetoid movements of the trunk and limbs. Her speech was incoherent, with frequent derailment and loss of logical connections. Affect was strikingly labile, oscillating between terrified agitation with recoil from staff and periods of flat resignation accompanied by self-deprecatory muttering. Thought content revealed alternating themes dominated by fixed paranoid delusions of contamination by staff and delusions of passivity ("they make me do this"). No overt hallucinations were reported. Cognitive assessment demonstrated gross impairment, with disorientation to time, place, and person. Formal testing of attention, memory, and executive function was impossible due to poor engagement. She exhibited a complete lack of insight and severely compromised judgment.

Given the potential involvement of neuropsychiatric lupus and concerns about chemotherapy-related neurotoxicity, collaboration with neurology and rheumatology was recommended. The rheumatology team conducted a comprehensive lupus workup, including tests for antinuclear antibody (ANA) enzyme-linked immunosorbent assay (ELISA), antineutrophil cytoplasmic antibodies (ANCA) proteinase 3 (PR3), ANCA myeloperoxidase (MPO), beta-2 glycoprotein antibodies, cardiolipin antibodies, complement component 3 (C3) and component 4 (C4), erythrocyte sedimentation rate (ESR), and a coagulation profile, all of which were within normal limits (Table 1). Routine blood work revealed no signs of infection or elevated inflammatory markers, indicating active disease.

**Table 1:** Results of routine blood tests and autoimmune workup conducted during initial assessment. AB: antibody; adjusted Ca: adjusted calcium (corrected for albumin); ALP: alkaline phosphatase; ALT: alanine aminotransferase; ANA ELISA: antinuclear antibody (via enzyme-linked immunosorbent assay); ANCA MPO: antineutrophil cytoplasmic antibody-myeloperoxidase; ANCA PR3: antineutrophil cytoplasmic antibody-proteinase 3; anti-B2GP1: anti-beta-2 glycoprotein 1; APTT: activated partial thromboplastin time; C3: complement component 3; C4: complement component 4; Ca: calcium; CRP: C-reactive protein; eGFR: estimated glomerular filtration rate; ESR: erythrocyte sedimentation rate; free T4: free thyroxine; Hb: hemoglobin; IgG: immunoglobulin G; IgM: immunoglobulin M; MCH: mean corpuscular hemoglobin; MCV: mean corpuscular volume; Mg: magnesium; Na: sodium; PT: prothrombin time; RBC: red blood cell count; RDH: red cell distribution width; TSH: thyroid stimulating hormone; WBC: white blood cell count

Investigation/tests	Values	Range
Ca	2.39 mmol/L	2.2-2.6 mmol/L
Adjusted Ca	2.36 mmol/L	2.2-2.6 mmol/L
Mg	0.97 mmol/L	0.7-1 mmol/L
TSH	0.64 mIU/L	0.27-4.2 mIU/L
Free T4	23.0 pmol/L	12-22 pmol/L
Vitamin B12	439 ng/L	197-771 ng/L
Serum folate	5.9 μg/L	3-17 μg/L
Albumin	44 g/L	35-50 g/L
Total protein	64 g/L	60-80 g/L
Globulin	20 g/L	20-35 g/L
Total bilirubin	6 μmol/L	0-20 μmol/L
ALP	75 U/L	30-130 U/L
ALT	20 U/L	0-33 U/L
Na	136 mmol/L	133-146 mmol/L
K	3.7 mmol/L	3.5-5.3 mmol/L
Urea	6.4 mmol/L	2.5-7.8 mmol/L
Creatinine	44 μmol/L	45-84 μmol/L
eGFR	>90 mL/min	-
ESR	11 mm/h	0-30 mm/h
CRP	2 mg/L	0-5 mg/L
Hgb	118 g/L	115-165 g/L
WBC	9.1×10^9^/L	4-11×10^9^/L
Platelets	475×10^9^/L	135-450×10^9^/L
RBC	3.75×10^12^/L	3.8-4.8×10^12^/L
Hematocrit	0.347 L/L	0.37-0.47 L/L
MCV	92.5 fL	80-100 fL
MCH	31.5 pg	27-34 pg
RDW	12.5%	10-15%
Neutrophils	6.18×10^9^/L	2.0-7.5×10^9^/L
Lymphocytes	2.05×10^9^/L	1.0-4.0×10^9^/L
Monocytes	0.81×10^9^/L	0.2-1.0×10^9^/L
Eosinophils	0.03×10^9^/L	0.0-0.5×10^9^/L
Basophils	0.04×10^9^/L	0.02-0.1×10^9^/L
PT	12.9 s	10-15 s
APTT	27.3 s	25-36 s
Fibrinogen	3.41 g/L	2-4.5 g/L
Immunology		
C3	1.50 g/L	0.9-1.8 g/L
C4	0.34 g/L	0.1-0.4 g/L
ANA ELISA	0.9 ratio	0.0-0.9 ratio
ANCA MPO	<0.2 IU/mL	0.0-3.4 IU/mL
ANCA PR3	<0.6 IU/mL	0.0-1.9 IU/mL
Anti-B2GP1 IgG AB	<1 U/mL	0-6 U/mL
Anti-B2GP1 IgM AB	<3 U/mL	0-6 U/mL
Cardiolipin IgG AB	2 GPL-U/mL	0-9 GPL-U/mL
Cardiolipin IgM AB	4 GPL-U/mL	0-9 GPL-U/mL

**Figure 1:**
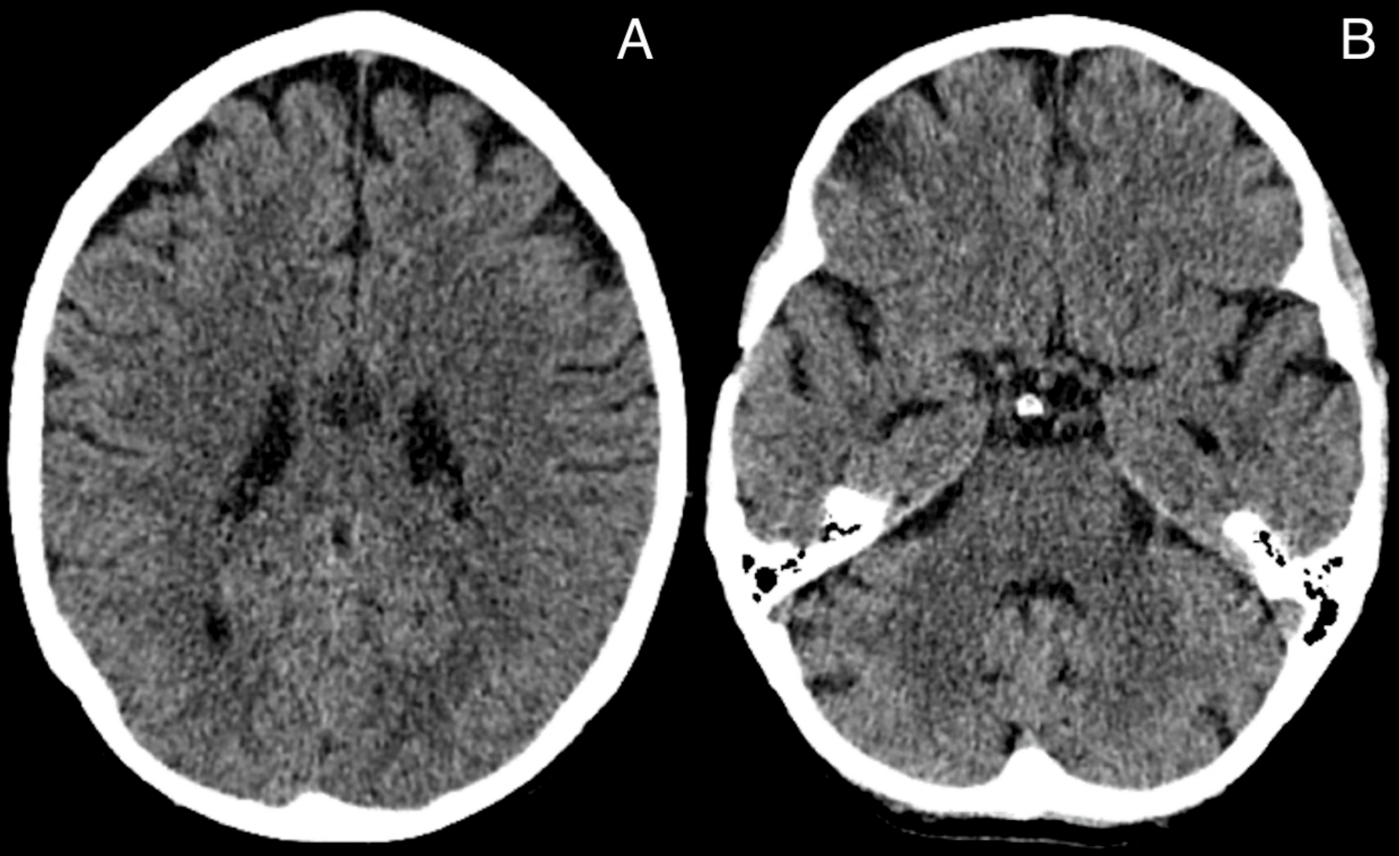
Non-contrast CT brain images showing no evidence of acute intracranial pathology. A: axial plane, demonstrating no evidence of acute intracranial pathology at the level of the cerebral cortex and basal ganglia. B: axial plane, demonstrating no evidence of acute intracranial pathology at the level of the tentorium cerebelli.

**Figure 2:**
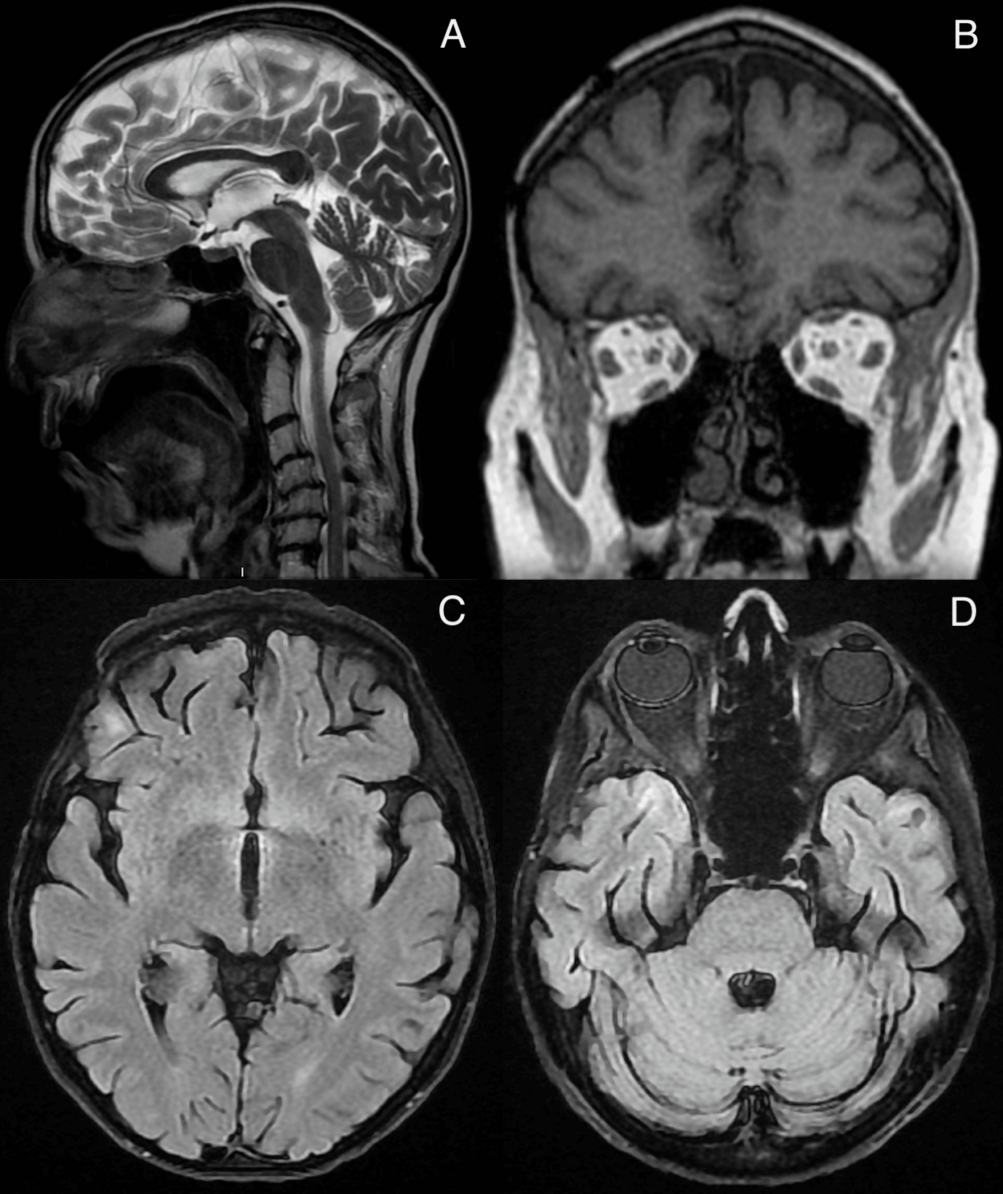
MRI brain sequences demonstrating no abnormal signal or structural lesions suggestive of active neuropsychiatric lupus or metastatic disease. A: sagittal plane, demonstrating no evidence of acute intracranial pathology at the level of midline. B: coronal plane, demonstrating no evidence of acute intracranial pathology at the level of the frontal lobes. C: axial plane, demonstrating no evidence of acute intracranial pathology at the level of the cerebral cortex and basal ganglia. D: axial plane, demonstrating no evidence of acute intracranial pathology at the level of the tentorium cerebelli.

Despite these normal findings, the team recommended increasing the dosage of prednisolone to 30 mg to address potential neuropsychiatric lupus involvement. During the neurologist’s assessment, the involuntary movements initially observed in the patient in the emergency department had disappeared. A CT scan was performed to evaluate possible structural causes, followed by an MRI and EEG of the brain (Figures 1A, 1B). Both investigations revealed no significant abnormalities, adding further complexity to the diagnostic process and raising questions about the underlying etiology of her symptoms (Figures 2A-2D).

To address the patient’s psychotic symptoms, olanzapine 2.5 mg orally twice daily was initiated, and lorazepam 0.5 mg orally three times daily was prescribed to manage anxiety and agitation. Her olanzapine was up-titrated to 5 mg twice daily and then to 5 mg in the morning and 7.5 mg at night, after a partial response was seen. Although her distress lessened, her paranoid delusions persisted, albeit at a reduced intensity.

## Discussion

This case highlights significant challenges associated with diagnosing a patient with overlapping neuropsychiatric symptoms, particularly in the presence of multiple comorbidities. A primary challenge was distinguishing between a primary psychiatric disorder and other neuropsychiatric conditions. The patient's clinical presentation, marked by both psychiatric and neurological features, strongly suggested an underlying secondary cause. However, the absence of abnormalities on routine investigations, including SLE and APS workup and brain MRI imaging, complicated the diagnostic process and necessitated further evaluation.

Given this diagnostic uncertainty, a comprehensive and multidisciplinary approach was essential. This case underscores the importance of continuous reassessment, targeted investigations, and appropriate referrals across specialties to refine the diagnostic formulation and optimize patient care. However, it is crucial to acknowledge that, in clinical practice, a definitive diagnosis may not always be attainable. In such cases, management should focus on symptom control, close monitoring, and a flexible treatment strategy that adapts to the evolving clinical picture.

Neuropsychiatric systemic lupus erythematosus (NPSLE)

The differential diagnosis was broad due to the patient's complex medical background. One key consideration was neuropsychiatric systemic lupus erythematosus (NPSLE), given the patient's presentation with psychosis, confusion, and involuntary movements. Although these symptoms can be consistent with NPSLE, the absence of active lupus on immunological testing and the normal MRI findings made this diagnosis less likely, though it could not be entirely excluded. NPSLE remains a clinical diagnosis, and laboratory findings may not necessarily reflect the full extent of the patient's presentation. Therefore, a careful and holistic approach is required to differentiate between a primary psychiatric disorder and a secondary neuropsychiatric condition like NPSLE.

Although brain MRI imaging is a common tool for investigating neuropsychiatric symptoms in lupus patients, a normal MRI does not rule out NPSLE. Clinicians emphasise that many neuropsychiatric symptoms of SLE often relapse and remit without detectable structural changes on imaging, yet these symptoms can still be directly attributed to the disease. It is important to stress that lupus can affect blood vessels and nerves throughout the body, even in the absence of visible changes on brain scans [5]. This underscores the complex and systemic nature of NPSLE, where neuropsychiatric symptoms can manifest even without structural abnormalities on MRI or other diagnostic tests [5]. Additionally, the initial presentation of movement symptoms, which later disappeared, despite the persistence of her psychiatric symptoms of agitation and paranoid delusions, also points toward NPSLE. It is possible that the disappearance of these symptoms was due to a higher dose of steroids, which is often used to manage inflammatory or autoimmune-related neurological symptoms.

Steroid-induced neuropsychiatric effects

Prednisolone, a corticosteroid commonly used to treat autoimmune conditions, is associated with a variety of neuropsychiatric complications, including mood disturbances, cognitive decline, and psychosis. These effects are particularly prevalent with long-term use or high doses, as the medication influences the central nervous system through alterations in neurotransmitter levels and immune system modulation. While neuropsychiatric side effects are more frequently observed in patients with pre-existing mental health conditions or those on prolonged steroid regimens, they can also occur in individuals without prior psychiatric history [6].

In this patient, prednisolone is being used to manage SLE. Despite a stable dose of 10 mg daily, which reduces the likelihood of recent dose changes contributing to the acute neuropsychiatric symptoms, corticosteroids can still provoke such effects. This complicates the differential diagnosis, as the symptoms may either result from the medication itself or from NPSLE [7].

Brain metastases and temporal lobe resection

Brain metastasis was initially considered as a potential diagnosis, especially given the patient's history of breast cancer with previous brain metastases. However, brain MRI findings revealed no significant abnormalities, reducing the likelihood of this diagnosis. Another important factor in this patient's presentation was her history of brain metastasis, which required a temporal lobe resection. The temporal lobes, particularly the medial temporal structures, play a crucial role in memory, emotional regulation, and higher cognitive functions. Disruption of these functions due to brain metastasis can contribute to both neurological and psychiatric symptoms [8].

Following temporal lobe resection, patients may experience changes in mood, cognition, and behavior, which can predispose them to psychiatric disorders. Lesions or surgical removal of the anterior temporal lobe, in particular, are known to lead to psychotic symptoms such as delusions, hallucinations, and disorganized thinking. These symptoms can be further exacerbated in patients with pre-existing brain pathology, like metastasis or neoplasms, where neurological function is already compromised [8]. Thus, the patient's history of temporal lobe resection following brain metastasis is likely a significant factor in the onset of her psychiatric symptoms, including psychosis.

Chemotherapy-induced neurotoxicity

Chemotherapy-induced neurotoxicity is a well-documented phenomenon that encompasses a spectrum of neuropsychiatric manifestations, including cognitive impairment, mood disturbances, and, in rare cases, psychosis. Case studies have reported instances of acute psychosis following chemotherapy, such as a documented case of a 42-year-old woman with triple-negative breast cancer who developed psychotic symptoms after her second cycle of neoadjuvant chemotherapy. While there is no definitive evidence implicating chemotherapy as the primary etiology in this case, its potential role as a contributing factor cannot be excluded, particularly given the patient's ongoing treatment regimen [9,10].

Paraneoplastic syndrome (PNS)

Paraneoplastic syndromes (PNS) are rare disorders that arise from indirect effects of malignancies, often preceding the cancer diagnosis and progressing independently of tumor activity. These syndromes can present with a variety of neuropsychiatric manifestations, including psychosis and movement disorders, due to immune-mediated effects on the central nervous system [11,12]. In this case, although the patient's evaluation did not reveal typical markers of PNS, such as anti-neuronal antibodies, the possibility remains, particularly given her history of breast cancer. Notably, the observed involuntary movements resolved following an increase in steroid dosage, suggesting a potential paraneoplastic etiology, as corticosteroids are a common therapeutic approach for managing PNS-related symptoms.

Primary psychiatric disorder

The possibility of a primary psychiatric disorder, such as a delusional disorder or a primary psychotic illness, was carefully considered. However, the patient’s complex medical history, the nature of her symptoms, and the presence of neurological manifestations suggested that an underlying medical or secondary cause was more plausible. Furthermore, the temporal relationship between her psychiatric symptoms and medical conditions, along with the absence of a well-defined progression typical of primary psychotic disorders, further supported the likelihood of a secondary etiology. Consequently, while a primary psychiatric disorder could not be entirely ruled out, it was considered less probable in this case.

## Conclusions

This case involved a psychotic patient with a broad differential diagnosis, including NPSLE, steroid-induced psychosis, brain metastasis, temporal lobe resection, chemotherapy-induced neurotoxicity, PNS, and primary psychiatric disorder. It demonstrates the difficulty of establishing a definitive diagnosis, given the patient's multifaceted clinical presentation and the presence of multiple comorbidities, particularly in the context of inconclusive investigative findings. A multidisciplinary approach in investigating and managing this case is imperative, especially when the patient’s comorbidities can contribute to overlapping neuropsychiatric symptoms. In this context, it is imperative that clinicians adopt a pragmatic and holistic approach to symptom management, emphasizing close monitoring and maintaining a flexible treatment strategy in response to the evolving clinical picture, rather than becoming overly fixated on achieving a definitive diagnosis.

## References

[1] Hanly JG, Urowitz MB, Su L (2010). Prospective analysis of neuropsychiatric events in an international disease inception cohort of patients with systemic lupus erythematosus. Ann Rheum Dis.

[2] Warrington TP, Bostwick JM (2006). Psychiatric adverse effects of corticosteroids. Mayo Clin Proc.

[3] Tchen N, Juffs HG, Downie FP (2003). Cognitive function, fatigue, and menopausal symptoms in women receiving adjuvant chemotherapy for breast cancer. J Clin Oncol.

[4] Caraceni A (2013). Drug-associated delirium in cancer patients. EJC Suppl.

[5] Sloan M, Andreoli L, Zandi MS (2024). Attribution of neuropsychiatric symptoms and prioritization of evidence in the diagnosis of neuropsychiatric lupus: mixed methods analysis of patient and clinician perspectives from the international INSPIRE study. Rheumatology (Oxford).

[6] Koning AC, van der Meulen M, Schaap D (2024). Neuropsychiatric adverse effects of synthetic glucocorticoids: a systematic review and meta-analysis. J Clin Endocrinol Metab.

[7] Peterson LG, Popkin MK (1980). Neuropsychiatric effects of chemotherapeutic agents for cancer. Psychosomatics.

[8] Shaw P, Mellers J, Henderson M, Polkey C, David AS, Toone BK (2004). Schizophrenia-like psychosis arising de novo following a temporal lobectomy: timing and risk factors. J Neurol Neurosurg Psychiatry.

[9] Garg H, Prakash S, Deb KS, Chadda RK (2018). Secondary mania following cancer chemotherapy with capecitabine. BMJ Case Rep.

[10] Alshehri S, Assiri H, Alsalem M, Alharbi MA (2022). Secondary psychosis following neoadjuvant AC-T chemotherapy for triple-negative breast cancer: case report and literature review of psychosis postchemotherapy. Case Rep Psychiatry.

[11] Lemos M, Lourenço A, Ribeiro M (2022). Psychiatric manifestations of paraneoplastic syndromes. Eur Psychiatry.

[12] Kayser MS, Kohler CG, Dalmau J (2010). Psychiatric manifestations of paraneoplastic disorders. Am J Psychiatry.

